# Relationship between the Depth of Acetabuloplasty and Outcomes of Hip Arthroscopy in Patients with Global Pincer Femoroacetabular Impingement: Study with a Minimum Follow‐Up Period of 2 Years

**DOI:** 10.1111/os.13739

**Published:** 2023-04-27

**Authors:** Xiao‐Dong Ju, Zi‐Yi He, Han‐Han Dang, Xin Zhang, Zhu Zhang, Yan Xu, Hong‐Jie Huang, Jian‐Quan Wang

**Affiliations:** ^1^ Department of Sports Medicine Peking University Third Hospital, Institute of Sports Medicine of Peking University Beijing China; ^2^ Beijing Key Laboratory of Sports Injuries Beijing China; ^3^ Engineering Research Center of Sports Trauma Treatment Technology and Devices Ministry of Education Beijing China

**Keywords:** Acetabular Rim Resection, Femoroacetabular Impingement, Global Pincer, Hip Arthroscopy

## Abstract

**Objective:**

There has been no definite consensus on the ideal depth of acetabuloplasty, especially in cases of global pincer femoroacetabular impingement (FAI). This study aims to determine whether the depth of acetabuloplasty influences postoperative outcomes in cases of global pincer FAI.

**Methods:**

Data were retrospectively collected from patients with global pincer FAI who underwent hip arthroscopy with a minimum follow‐up period of 2 years from May 2014 to December 2018. Patients with global pincer FAI were subdivided into low or high resection depth groups based on whether the intraoperative acetabular rim was resected by more than 3 mm. Radiographic measurements; arthroscopic procedures; preoperative and postoperative PROs were recorded. Achievement of MCID and PASS was compared for the VAS, mHHS, HOS‐ADL, and iHOT‐12. A paired Student t‐test was used to evaluate the significance of preoperative and postoperative PROs and two‐tailed unpaired Student t‐test was used to compare demographic data and PROs between different groups. MCID and PASS were evaluated using the chi‐square test or the Fisher's exact test.

**Results:**

A total of 41 hips with global pincer FAI (15 and 26 patients in low or high resection depth groups, respectively) were included in this study. Both groups showed significant postoperative improvements in the scores of all PROs (p < 0.001). Compared to the low resection depth group, the high resection depth group had a lower degree of improvement through hip arthroscopy, which manifested as lower postoperative mHHS scores (94.29 vs*.* 85.08, *p* = 0.006), higher VAS scores (0.93 vs*.* 2.54, *p* = 0.002), and lower improvements in VAS (−5.00 vs*.* −3.35, *p* = 0.028), HOS‐ADL (34.99 vs*.* 23.90, *p* = 0.017) and iHOT‐12 (39.89 vs*.* 29.27, *p* = 0.036). Patients in high resection depth group were less likely to achieve the MCID for the VAS score compared to low resection depth group in significant (73.3 vs*.* 26.9%, *p* = 0.004).

**Conclusions:**

For patients with global pincer, the outcomes in high resection depth group were slightly worse than the the low resection depth group. It is indicated that excessive resection of the acetabular rim during the procedure should be avoided.

## Graphical abstract

To determine whether the depth of acetabuloplasty influences outcomes in cases of global pincer FAI. The results of our study suggest that excessive resection of the acetabular rim during the procedure should be avoided.

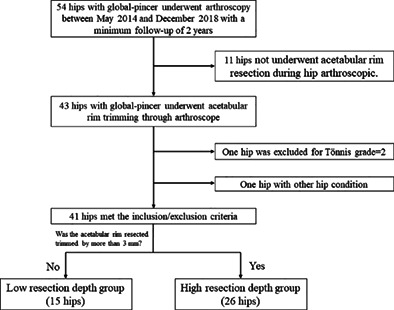

## Introduction

Femoroacetabular impingement (FAI) is a condition that causes hip pain and dysfunction with the subsequent development of primary osteoarthritis.^1^ There are three types of FAI: cam, pincer, and mixed.^2^ Cam‐type impingement is caused by abnormal or aspheric morphology of the femoral head, and pincer‐type impingement is caused by overcoverage of the acetabulum.^3^, ^4^, ^5^ Most patients with FAI often have both components.^6^ Pincer FAI occurs in the focal or global form.^7^ Compared to the former, the latter is relatively rare in clinical settings; however, the symptoms are often more serious due to a broader range of acetabular overcoverage. Patients with global pincer FAI usually have a lateral center‐edge angle (LCEA) > 40° and a Tönnis angle <0° with the finding of coxa profunda or acetabular protrusion on radiography.^7^, ^8^, ^9^

Earlier, patients with global pincer FAI were recommended to undergo open surgery.^8^, ^10^ However, increasing evidence in recent years has shown that arthroscopic treatment for patients with global pincer FAI is safe and effective.^8^, ^9^, ^11^ For patients with pincer‐type FAI, the usual treatment is acetabular rim trimming to resect lesions.^12^ A previous study showed that arthroscopic treatment for global pincer FAI could afford results as good as those obtained in patients with focal pincer FAI, although it was a cohort study consisting of a small number of patients.^13^ While another study found that hips with lateral overcoverage combined with coxa profunda have a smaller potential for improvement compared with hips with normal coverage.^14^ There is a view that this contradictory conclusion should be attributed to higher intraoperative resection of the acetabulum.^15^ A previous study reported that there was no correlation between the depth of acetabuloplasty or postoperative LCEA and the outcomes of hip arthroscopy.^16^ However, it did not control the preoperative LCEA of patients and the results of the study have been controversial.^17^ Because of the broader range of acetabular overcoverage in cases of global pincer FAI, patients usually undergo more extensive resections. Some operators reduced the LCEA to approximately 35° according to their experience.^13^ Thus far, there has been no consensus on the ideal depth of acetabuloplasty, especially in cases of global pincer FAI. Thus, this study aimed to (i) determine whether arthroscopy can be used to treat global pincer and achieve good results and (ii) evaluate the relationship between the extent of pincer resection and the prognosis of patients with global pincer FAI. We hypothesized that patients with global pincer FAI would have favorable postoperative outcomes and that we could determine the optimum depth of acetabuloplasty for patients with global pincer FAI.

## Methods

### Patient Selection

**FIGURE 1:**
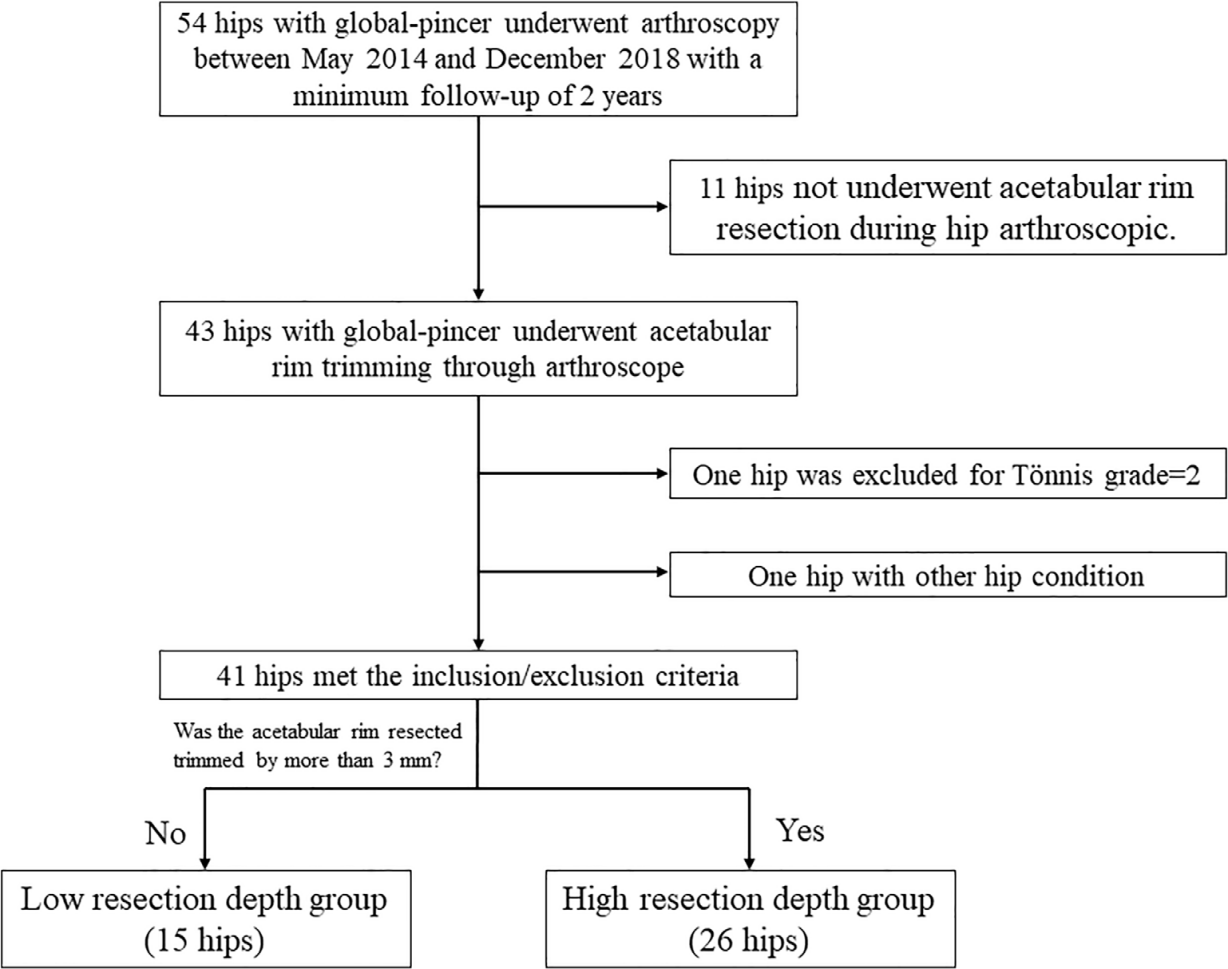
Patient selection flowchart.

This study was approved by the Peking University Third Hospital Medical Science Research Ethics Committee (M2019193). Data were retrospectively analyzed from May 2014 to December 2018. Patients were included in the global pincer cohort if they (1) were diagnosed with FAI and underwent unilateral hip arthroscopy without other hip conditions; (2) had a LCEA of ≥40° and showed coxa profunda or acetabular protrusion on radiography; and (3) were followed up for a minimum of 2 years. Patients were excluded if they had moderate‐to‐advanced osteoarthritis (Tӧnnis grade >1). Patients who underwent acetabuloplasty and labral repair at the 1 to 3 o'clock position without acetabuloplasty at the 12 o'clock position were also excluded, because they had no labral tear and cartilage injury at the 12 o'clock position (Figure 1).

### Radiographic Measurements and Grouping

**FIGURE 2:**
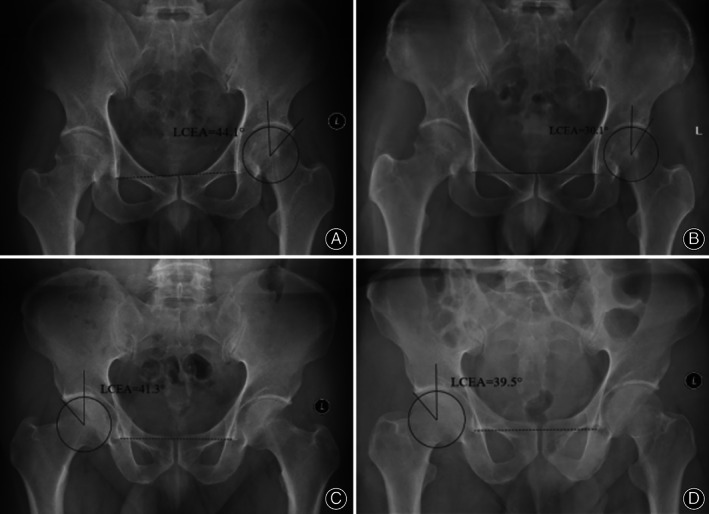
Preoperative and postoperative AP radiographs of the pelvis in the high (A and B) and low (C and D) resection depth groups. LCEA, lateral center‐edge angle.

All the patients underwent preoperative and postoperative AP radiography of the pelvis and 45° Dunn radiography. The LCEA angle and Tӧnnis grade were measured on AP pelvis radiographs, and the alpha angle was measured on 45° Dunn radiographs. Preoperative and postoperative LCEAs were measured in all the patients with global pincer FAI included in the study, and intraoperative changes were calculated. The coronal plane in X‐ray was determined by the line that connected inferior border of the acetabular tear drops bilaterally. Place the most appropriate circle in the femoral head and take the center of the circle as the center point of the femoral head to measure LCEA (Figure 2). According to the method proposed by Philippon, the extent of changes in the LCEA via intraoperative fluoroscopy could be used to estimate the degree of acetabular rim resection at the 12 o'clock position on the acetabular surface during arthroscopy. The following formula was used for calculation: change in the LCEA = 1.8 + (0.64 × rim reduction in millimeters).^18^ According to the extent of acetabular rim resection calculated using the formula, patients with global pincer FAI were further divided into two groups: low and high resection depth groups. The criterion for this division was whether the acetabular rim was resected by more than 3 mm, that is, the LCEA was reduced by 3.7°, which was jointly determined by the senior authors based on their clinical experience.

### Patient‐Reported Outcomes

Patient‐reported outcomes (PROs) including the modified Harris Hip Score (mHHS), Hip Outcome Score‐Activities of Daily Living (HOS‐ADL), and International Hip Outcome Tool 12‐component form (iHOT‐12) scores before and after surgery were used to evaluate the clinical outcomes of arthroscopic treatment. Furthermore, preoperative and postoperative visual analog scale (VAS) scores were also used for pain assessment. Postoperative satisfaction was evaluated based on patient reports. The minimal clinically important difference (MCID) and Patient Acceptable Symptomatic State (PASS) of mHHS, VAS, HOS‐ADL, and iHOT‐12 were determined and compared between groups. According to authoritative literature reports and following some previous studies, the MCID was defined as a change of 8, 1.5, 9, and 13 points in the mHHS and VAS, HOS‐ADL, and iHOT‐12 scores, respectively^19^, ^20^, ^21^; and the PASS was defined as the achievement of 74, 1.91, 87, and 72.2 points for mHHS, VAS, HOS‐ADL, and iHOT‐12, respectively.^19^, ^21^, ^22^, ^23^

### Surgical Procedures

All hip arthroscopies were performed by three senior authors. After anesthesia, the patient was placed in the modified supine position on standard hip traction (Smith & Nephew). Three standard portals were used: anterolateral (AL) portal, mid‐anterior portal (MAP), and proximal mid‐anterior portal (PMAP). Most pathologies in the central compartment, including the pincer deformity, labral injury, and cartilage injury, can be treated with the AL portal and MAP. The labrum treatment, including debridement, repair, or reconstruction, was recorded during surgery. After the addressing pathology in the central compartment, the arthroscope was introduced into the peripheral compartment for osteochondroplasty of the cam deformity by a high‐speed burr (Smith & Nephew American). Finally, the incised joint capsule was repaired routinely before closure.

### Postoperative Rehabilitation

The patient mainly rested in bed and raised the affected limb on the first day after the operation. From the first day to the second, the patient began isometric contraction exercises comprising ankle pumps and exercises of the quadriceps femoris and muscles around the hip joint under guidance. From the third day to the seventh, patients walked with a partial weight on the affected limb and started a passive hip movement within the painless range. Partial weight‐bearing was conducted 4–6 weeks after the operation, and active hip joint activities within the tolerable range were carried out, including adduction, abduction, and internal and external rotation, and exercises for strengthening of hip abduction, flexion, and extension, while continuing passive hip joint activities. After 6 weeks, the patients could walk with a full load and recovered normal functional activities of the lower limbs. From the third to the sixth month after the operation, the patients gradually returned to normal activity levels and tried sports.

### Statistical Analysis

All data were first assessed for normal distribution and homogeneity of variance by using the Shapiro–Wilk and Fisher tests. Then, a two‐tailed unpaired Student t‐test was used to compare demographic data and PROs between different cohorts. The two‐tailed paired Student t‐test was used to evaluate the significance of preoperative and postoperative PROs. Categorical data were evaluated using the chi‐square test or the Fisher's exact test. All data were statistically analyzed using the SPSS 26.0 (IBM, Armonk, NY) software and are expressed as mean ± standard deviation values. *p*‐values < 0.05 were considered statistically significant.

## Results

### Patient Demographics

Overall, 41 patients with global pincer FAI were included in this study. The demographic and radiographic data of the two groups are summarized in Table 1. According to whether the acetabular rim was resected by more than 3 mm (LCEA reduced by approximately 3.7°), 41 patients with global pincer FAI were divided into the low and high resection depth groups (15 and 26 patients, respectively). There were no significant intergroup differences in preoperative LCEA and α angle, age, sex, BMI, side of the hip, or follow‐up time. In particular, there was no difference in the postoperative α angle between the two groups. In the low resection depth group, LCEA was reduced from 44.12 ± 2.39 to 42.48 ± 2.20, while in the high resection depth group, it was reduced from 44.16 ± 3.80 to 35.58 ± 4.39 (*p* < 0.001).

**TABLE 1:** Patient demographic and radiographic characteristics for the low and high resection depth groups in the global pincer group.^a^

Category	Low resection depth (15 hips)	High resection depth (26 hips)	*p* value
Age, years	37.07 ± 11.06	40.19 ± 8.78	0.324
Body mass index, kg·m^−2^	24.29 ± 3.25	23.66 ± 3.15	0.558
Sex, male/female	10/5	14/12	0.422
Side involved, right/left	5/10	11/15	0.570
Preoperative α angle, deg	62.91 ± 4.40	64.77 ± 8.21	0.352
Postoperative α angle, deg	43.03 ± 3.31	43.15 ± 3.85	0.915
Change in α angle, deg	19.87 ± 5.96	21.62 ± 8.27	0.483
Preoperative LCEA, deg	44.12 ± 2.39	44.16 ± 3.81	0.970
Postoperative LCEA, deg	42.48 ± 2.20	35.58 ± 4.39	**<0.001**
Change in LCEA, deg	1.64 ± 0.94	8.58 ± 3.82	**<0.001**
Follow up, mo	38.87 ± 11.78	40.92 ± 11.10	0.579

^a^ Values are presented as mean ± SD unless noted otherwise. LCEA, lateral center‐edge angle. Bold indicates statistical significance (*p* < 0.05).

### Procedures Performed

All patients underwent acetabular rim trimming according to the inclusion criteria and all patients in both of the groups underwent femoroplasty due to the abnormal morphology of the femoral head (cam FAI). There were no significant differences in other intraoperative variables between the two groups (Table 2).

**TABLE 2:** Intraoperative procedures for the low and high resection depth groups in the global pincer group.^a^

Category	Low resection depth (15 hips)	High resection depth (26 hips)	*p* value
Labral repair			
Debridement	0 (0)	2 (7.7)	0.695
Repair	15 (100)	23 (88.5)	
Reconstruction	0 (0)	1 (3.8)	
Acetabuloplasty	15 (100)	26 (100)	>0.999
Femoroplasty	15 (100)	26 (100)	>0.999
Femoral head chondroplasty	2 (13.3)	2 (7.7)	0.968
Synovectomy	15 (100)	24 (92.3)	0.396
Trochanteric bursectomy	5 (33.3)	9 (34.6)	0.934
Iliopsoas fractional lengthening	4 (26.7)	4 (15.4)	0.639

^a^ Values are presented as n (%).

### Patient‐Reported Outcomes

**FIGURE 3:**
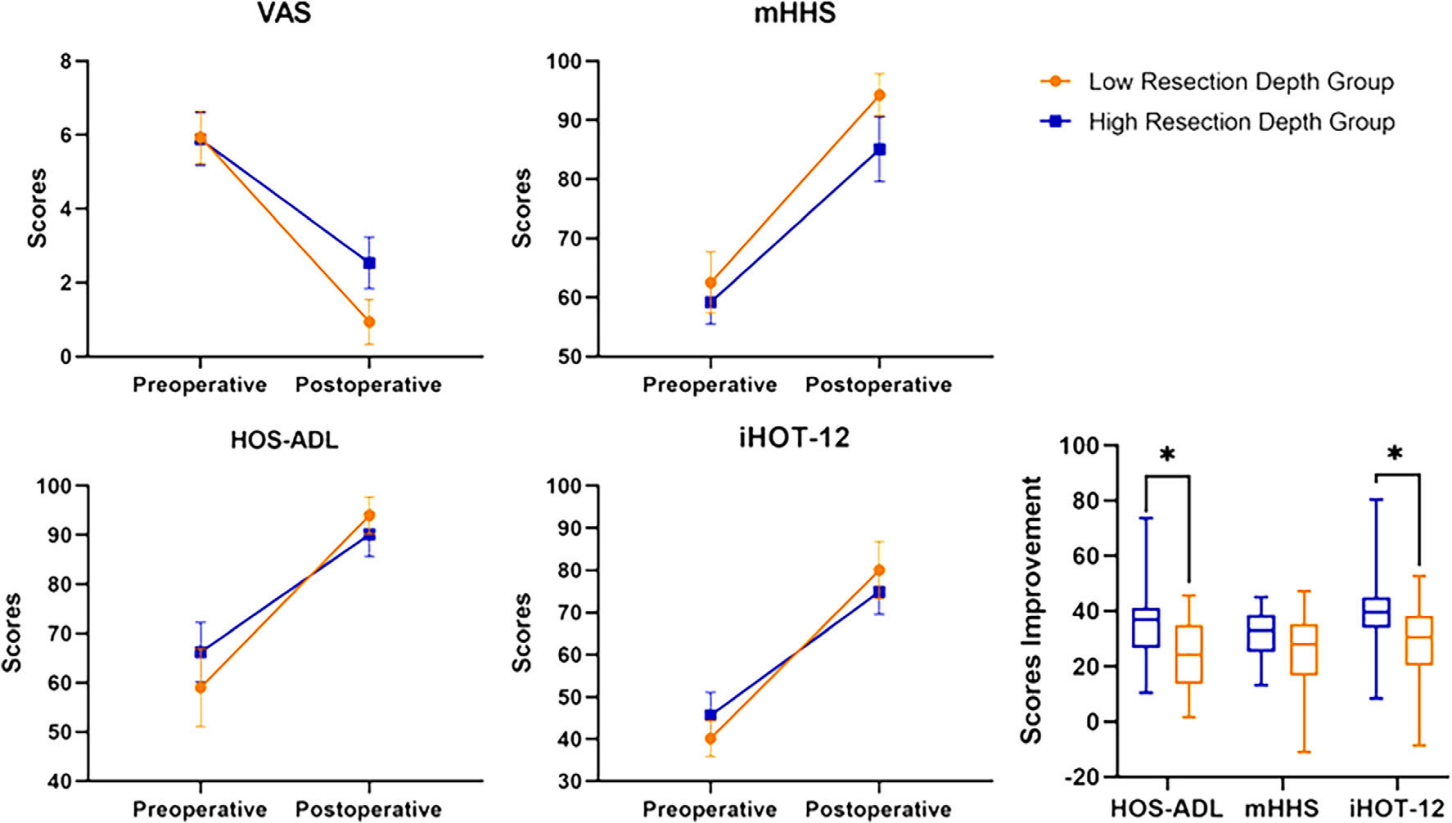
Pre‐ and postoperative outcome scores in the low and high resection depth groups. Error bars are 95% Cis.

No significant differences were observed in the preoperative scores for the VAS, mHHS, HOS‐ADL, and iHOT‐12 between the different resection depth groups. The scores of all the PROs at the final follow‐up were significantly better than the preoperative levels (*p* < 0.001 for all) in both groups (Table 3). In comparison with the low resection depth group, the high resection depth group showed lower postoperative mHHS scores (94.29 vs. 85.08, *p* = 0.006), higher VAS pain scores (0.93 vs. 2.54, *p* = 0.002). Meanwhile, the improvements in VAS (−5.00 vs. −3.35, *p* = 0.028), HOS‐ADL (34.99 *vs*. 23.90, *p* = 0.017), and iHOT‐12 (39.89 *vs*. 29.27, *p* = 0.036) scores were lower in high resection depth group (Table 3 and Figure 3).

**TABLE 3:** Preoperative and postoperative patient‐reported outcome scores for the low and high resection depth groups.^a^

Category	Low resection depth (15 hips)	High resection depth (26 hips)^b^	*p* value
mHHS			
Preoperative	62.56 (57.36–67.77)	59.21 (55.49–62.94)	0.273
Postoperative	94.29 (90.74–97.84)	85.08 (79.61–90.54)	**0.006**
*p* value	**<0.001**	**<0.001**	
Improvement	31.72 (26.79–36.65)	25.87 (20.57–31.16)	0.133
VAS			
Preoperative	5.93 (5.22–6.64)	5.88 (5.17–6.60)	0.926
Postoperative	0.93 (0.32–1.54)	2.54 (1.84–3.24)	**0.002**
*p* value	**<0.001**	**<0.001**	
Improvement	−5.00 (−6.11–3.89)	−3.35 (−4.30–2.39)	**0.028**
HOS‐ADL			
Preoperative	59.02 (51.14–66.90)	66.23 (60.23–72.24)	0.132
Postoperative	94.02 (90.22–97.82)	90.13 (85.65–94.62)	0.216
*p* value	**<0.001**	**<0.001**	
Improvement	34.99 (26.98–43.01)	23.90 (18.46–29.34)	**0.017**
iHOT‐12			
Preoperative	40.13 (35.85–44.40)	45.60 (40.16–51.03)	0.147
Postoperative	80.02 (73.18–86.86)	74.87 (69.59–80.14)	0.216
*p* value	**<0.001**	**<0.001**	
Improvement	39.89 (31.77–48.02)	29.27 (22.97–35.57)	**0.036**

Abbreviations: HOS‐ADL, hip outcome scored‐activities of daily living; IHOT‐12, international hip outcome tool 12‐component form; LCEA, lateral center‐edge angle; mHHS, modified Harris Hip Score; VAS, visual analog scale.

^a^ Values are given as mean (95% Confidence Interval). Bold indicates statistical significance (*p* < −0.05).

^b^ PROs of HOS‐ADL and iHOT12 were missing in two patients of the high resection depth group.

Patients in high resection depth group were significantly less likely to achieve the MCID for the VAS score (73.3 vs. 26.9%, *p* = 0.004). However, there were no other differences in the rate of meeting the PASS and MCID between the low and high resection depth group despite similar trends (Table 4).

**TABLE 4:** Rates of MCID and PASS for the low and high resection depth groups.^a^

Category			Low resection depth (15 hips)	High resection depth (26 hips)^b^	*p* value
mHHS	MCID		15(100)	24(92.3)	0.524
	PASS		14(93.3)	22(84.6)	0.744
VAS	MCID		14(93.3)	20(76.9)	0.361
	PASS		11(73.3)	7(26.9)	**0.004**
HOS‐ADL	MCID		15(100)	22(91.7)	0.514
	PASS		13(86.7)	17(70.8)	0.437
iHOT‐12	MCID		14(93.3)	21(87.5)	>0.999
	PASS		11(73.3)	15(62.5)	0.728

Abbreviations: MCID, minimal clinically important difference; PASS, patient acceptable symptomatic state (PASS).

^a^ Values are presented as n (%). Bold indicates statistical significance (*p* < −0.05).

^b^ MCID and PASS of HOS‐ADL and iHOT12 were missing in two patients of the high resection depth group.

## Discussion

The main findings of this study were that patients with global pincer FAI can obtain clinically significant outcomes with arthroscope followed by a minimum follow‐up of 2 years. The postoperative PROs and improvement in high resection depth group were slightly worse than the the low resection depth group, which might indicate that the excessive resection of the acetabular rim during the procedure should be considered with caution.

### The Relationship between the Extent of Pincer Resection and the Arthroscopic Outcome

Acetabular overcoverage and coxa profunda increase the technical difficulty of arthroscopy and pose technical challenges to the operator. Although lateral acetabular overcoverage combined with coxa profunda can still be repairable with arthroscopic treatment, it shows smaller potential for improvement in comparison with hips showing normal coverage.^14^ Matsuda et al. and Sanders et al. reported that arthroscopic treatment of global pincer can achieve the same good outcome as that of focal pincer.^9^, ^13^ However, Chandrasekaran et al. compared the arthroscopic outcomes of patients with pincer‐type FAI who had a large LCEA (45.0 ± 4.69) and a hip arthroscopy control group with a normal LCEA (35.0 ± 7.52). They found that the former group showed a lower change in all PRO scores in comparison with the latter, as well as the satisfaction score.^14^ A similar study with a larger sample size and longer follow‐up time was conducted by Brick et al.^15^ Interestingly, they did not find the differences reported by Chandrasekaran et al.^14^ In addition, the rate of total hip arthroplasty in the pincer group in their study (1.2%) was lower than that reported by Chandrasekaran et al. (11.1%). Brick et al. explained that lesser resection of the acetabulum and a higher postoperative LCEA might be the reasons for the better results and lower conversion rates. Therefore, we wondered whether the amount of pincer resection would influence the outcomes of arthroscopy and developed a hypothesis. The results showed that the high resection depth group (>3 mm) had poorer outcomes than the low resection depth group, although only some of the postoperative scores for the PROs showed significant differences. Our hypothesis was supported by our findings. In the low resection depth group, LCEA was reduced from 44.12 ± 2.39 to 42.48 ± 2.20, while in the high resection depth group, it was reduced from 44.16 ± 3.80 to 35.58 ± 4.39. Both postoperative LCEA and rim reduction in millimeters showed significant differences (*p* < 0.001). The benefit of pincer resection in hip arthroscopy has been well‐received, but there are no reference standards for the extent of resection. Nevertheless, the findings confirm that greater resection does not necessarily yield better treatment effects.

### Potential Biomechanical Explanations for Excessive Resection

Colvin et al. performed open acetabular rim resection on 10 cadaveric hips. When they performed 4 mm of acetabular rim resection, 30% of the hips showed subluxation. Moreover, when they performed 5 mm of acetabular rim resection, 70% of the hips showed subluxation.^24^ Bhatia et al. found that acetabular rim resection of more than 4 to 6 mm increased hip joint contact pressures by three‐fold, and changed the contact pressure profile from a three‐point rim loading pattern to increased loading at the base of the acetabulum, they also found that excessive rim resection might lead to premature joint degeneration.^25^ In our study, 88% of the patients (23 of the 26 patients) in high resection depth group underwent resection of more than 4 mm of bone. Therefore, we speculated that excessive rim resection might cause the increased contact pressures, which lead to the different patient outcomes in the two groups.

We also speculated that another reason for this difference could be the role of the suction seal of the acetabular labrum. The acetabular labrum can enhance the distractive stability of the hip, prevent supraphysiologic distraction of the hip and regulate synovial fluid circulation.^26^, ^27^, ^28^ Suppauksorn et al. found that labral reconstruction could result in loss of suction seal,^29^ while Slullitel et al. thought that maintenance of the chondrolabral union can protect the function of suction seal and help improve stability, load distribution, and regulation of the synovial fluid.^30^ In the low resection depth group, the labrum was not completely detached, leaving the labrum and cartilage junction, which could maintain the role of the suction seal of the acetabular labrum. In comparison with the low resection depth group, the acetabulum in the high resection depth group was removed more and it must be completely detached from the acetabular rim during the operation. In that case, it may not be possible to fully restore the function of suction seal of the acetabular labrum after refixation, which could be another reason for the differences between low and high resection depth groups.

Johannsen et al. previously reported that there was no correlation between the depth of acetabuloplasty or postoperative LCEA and the outcomes of hip arthroscopy.^16^ On the basis of their results, the authors proposed that resection beyond the normal reference ranges was necessary to excise the nonfunctional bone to a stable base. Because the areas resected in acetabular rim trimming were not providing structural support preoperatively, these resections were less likely to significantly change the acetabular joint forces. In comparison with our study, the hip conditions of patients in their study were more complex. Three of the four groups of patients suffered from dysplasia, borderline dysplasia, borderline overcoverage/pincer and showed significant differences in preoperative LCEA (range, from <20° to >35°) between the groups. In our study, exploration of the extent of resection focused on the patients only with global pincer, and there was no significant difference in preoperative LCEA between the two groups (*p* = 0.830), which we speculated was the reason for the different conclusions from Johannsen et al.

### Excessive Rim Resections for Patients Should be Considered with Caution

In our study, we did not encourage operators to perform excessive rim resections for patients with a global pincer. After reviewing the relevant literature on global pincer, Coughlin et al. proposed that although a CT‐based analysis reported normal CEA was 31°, LCEA could be an individualized radiography index.^31^ This suggests that we should fully consider the condition of hip joint in every patient before acetabular rim trimming. After all, both overcorrection and undercorrection of femoroacetabular impingement in patients can lead to osseous deformities secondary.^32^ Due to the small number of patients in the global pincer cohort, our study could not determine a specific and suitable extent of acetabular rim resection during arthroscopy, if such a threshold actually exists. We recommend further research in order to bring more benefit for patients through acetabuloplasty.

### Strengths and Limitations

This study included more patients with global pincer and directly compared the preoperative and postoperative outcomes of hip arthroscopy. To our knowledge, this is the first study to discuss the influence of resection depth on postoperative pain relief and motor function of the hips in patients with a global pincer.

Our study had several limitations. First, the number of patients with global pincer in our study were small. We are concerned that they may not be representative of all patients. Second, the postoperative HOS‐ADL and iHOT‐12 scores in two patients with global pincer underwent high level acetabular rim resection could not be acquired, but we still included them for they had usable postoperative VAS and mHHS scores.

### Conclusion

Patients with global pincer FAI can obtain clinically significant outcomes with arthroscope followed by a minimum follow‐up of 2 years. In addition, our study found that patients underwent low level acetabular rim resection can acquired better postoperative improvements in pain relief and motor function compared with patients underwent high level acetabular rim resection.

## Author's Contribution

Xiao‐Dong Ju: Drafting the work and revising it critically for important intellectual content Zi‐Yi He: Drafting the work. Han‐Han Dang: Interpretation of data. Xin Zhang: Design of the work and revising it critically. Zhu Zhang: Collection and assembly of data. Yan Xu: Design of the work. Hong‐Jie Huang: Revising it critically for important intellectual content. Jian‐Quan Wang: Design of the work. All authors: Final approval of the version to be published.

## Conflict of Interest Statement

There are no conflicts of interest to declare.
